# Association of Cadmium, Lead and Mercury with Paraoxonase 1 Activity in Women

**DOI:** 10.1371/journal.pone.0092152

**Published:** 2014-03-28

**Authors:** Anna Z. Pollack, Lindsey Sjaarda, Katherine A. Ahrens, Sunni L. Mumford, Richard W. Browne, Jean Wactawski-Wende, Enrique F. Schisterman

**Affiliations:** 1 Division of Intramural Population Health Research, Eunice Kennedy Shriver National Institute of Child Health & Human Development, National Institutes of Health, Bethesda, Maryland, United States of America; 2 Department of Global and Community Health, School of Health and Human Services, George Mason University, Fairfax, Virginia, United States of America; 3 Department of Biotechnical and Clinical Laboratory Sciences, University at Buffalo, SUNY, Buffalo, New York, United States of America; 4 Department of Social and Preventive Medicine, University at Buffalo, SUNY, Buffalo, New York, United States of America; Stony Brook University, Graduate Program in Public Health, United States of America

## Abstract

**Background:**

The activity of paraoxonase 1 (PON1), an antioxidant enzyme whose polymorphisms have been associated with cancer risk, may be associated with metals exposure.

**Objective:**

To evaluate PON1 activity in relation to cadmium, lead, and mercury levels in healthy, premenopausal women.

**Methods:**

Women from upstate New York were followed for ≥ two menstrual cycles. Repeated measures linear mixed models estimated the association between cadmium, lead, and mercury levels (by tertile: T1, T2, T3) and PON1 arylesterase (PON1A) and PON1 paraoxonase (PON1P) activity, separately. Analyses were stratified by PON1 Q192R phenotype and un-stratified.

**Results:**

Median blood cadmium, lead, and mercury concentrations were 0.30 µg/L, 0.87 µg/dL, and 1.15 µg/L. In un-stratified analyses cadmium and mercury were associated with decreased PON1A activity (T2 vs. T1; not T3 vs. T1) but metals were not associated with PON1P. Phenotypes were distributed between QQ (n = 99), QR (n = 117), and RR (n = 34). Cadmium was associated with decreased PON1A activity for QR and RR phenotypes comparing T2 vs. T1 (−14.4% 95% confidence interval [CI] [−20.1, −8.4] and −27.9% [−39.5, −14.0],). Lead was associated with decreased PON1A (RR phenotype, T3 vs. T1 −18.9% [−32.5, −2.5]; T2 vs. T1 −19.6% [−32.4, −4.4]). Cadmium was associated with lower PON1P comparing T2 vs. T1 for the RR (−34.9% [−51.5, −12.5]) and QR phenotypes (−9.5% [−18.1, 0.0]) but not comparing T3 vs. T1. Cadmium was associated with increases in PON1P levels (QQ phenotype, T3 vs. T1 24.5% [7.0, 44.9]) and mercury was associated with increased PON1A levels (QQ phenotype, T3 vs. T1 6.2% [0.2, 12.6]). Mercury was associated with decreased PON1P (RR phenotype, T2 vs. T1 −22.8 [−37.8, −4.1]).

**Conclusion:**

Blood metals were associated with PON1 activity and these effects varied by phenotype. However, there was not a linear dose-response and these findings await replication.

## Introduction

Oxidative stress and free radicals are implicated in chronic disease development. Human serum paraoxonase 1 (PON1) is a key antioxidant enzyme that seems to play a role in carcinogenesis and cardiovascular disease [1], [2]. PON1 is associated with high density lipoprotein particles (HDL) that contributes to HDL's ability to reduce low density lipoprotein (LDL) lipid peroxidation [3]. Two PON1 gene polymorphisms confer greater or lesser protection from environmental toxicants [4], [5]. The polymorphism at amino acid position 192 (containing arginine [R] vs. glutamine [Q]) gives rise to 3 genotypic possibilities (RR, RQ or QQ), each associated with differing PON1 activities. For instance, PON1 hydrolytic activity against the substrate paraoxon (for which the enzyme was named) is lowest in QQ individuals and highest in those with RR, whereas protection against LDL oxidation appears greatest in QQ individuals and lowest in RR, with heterozygotes having intermediate enzyme activity for each [4], [6].

Environmental exposure to nonessential metals, such as cadmium, lead, and mercury, is a public health concern. Cadmium has been identified as a carcinogen by the International Agency for Research on Cancer [7]–[9], lead as a probable human carcinogen [10], and methylmercury as a possible carcinogen [11]. Cadmium and lead have been associated with increased risk of cardiovascular disease and mortality [12], peripheral arterial disease and chronic kidney disease [13], [14], while mercury has been associated with myocardial infarction but not hypertension or cardiovascular disease [15]–[17]. Metals may impact cardiovascular disease risk by increasing oxidative stress, including blood lipid peroxidation [18], a key component of the development of atherogenesis [19]. However, among young women, metal exposure was not associated with increased lipid peroxidation [20]. Nevertheless, metal exposure may depress lipid-associated enzyme function, thought to protect against lipid peroxidation, with implications for cardiovascular disease (CVD) risk with the progression of age and cumulative exposure. Further, PON has been proposed as a treatment target for atherosclerosis [21].

PON1 activity may be modulated by nonessential metal exposure and this effect might differ by PON1 phenotype [22]. Collectively, these data indicate a potential mechanism that could explain the association between metal exposure and cardiovascular disease risk [23]. However, data are lacking to determine the effects of blood concentrations of metals and the activity of HDL-associated PON1, and previous reports call for further study [6]. We hypothesized that blood metal levels would be associated with decreased PON1 antioxidant activity. Therefore, our study evaluated the association between blood levels of cadmium, lead, and mercury and PON1 activities by PON1 phenotype in a cohort of healthy, premenopausal women.

## Methods

### Study Cohort

The BioCycle Study enrolled healthy, premenopausal female volunteers (ages 18–44) to study the association between biomarkers of oxidative stress and hormone levels over the course of a regular menstrual cycle. Study inclusion criteria included a self-reported menstrual cycle length between 21 and 35 days for the past six months, not actively trying to conceive, no recent pregnancy or breastfeeding, and no history of polycystic ovary syndrome [24]. Women were followed prospectively for one (n = 9) or two (n = 250) menstrual cycles between 2005 and 2007. The study was conducted at the University at Buffalo in western New York State, under an Intramural Research Program contract from the *Eunice Kennedy Shriver* National Institute of Child Health and Human Development. The University at Buffalo Health Sciences Institutional Review Board (IRB) approved the study, and served as the IRB designated by the National Institutes of Health for this study under a reliance agreement. All participants provided written informed consent.

### Clinic Visits

At screening, women provided information on health and reproductive history, lifestyle information, and trained staff recorded height (meters) and weight (kilograms) to determine body mass index (BMI) (kg/m^2^). Whole food (fish, shellfish, vegetables, and grains) and nutrient intakes were assessed using the general food frequency questionnaire (FFQ) developed by the Nutrition Assessment Shared Resource of the Fred Hutchinson Cancer Center (Seattle, WA) at baseline using a 6 month recall. Women attended clinic visits to provide blood samples up to 8 times per menstrual cycle; corresponding to early menstruation (1 sample), mid- follicular phase (1 sample), expected time of ovulation (3 samples) and luteal phase (3 samples). Visits were scheduled to occur in the morning to obtain fasting blood samples and reduce diurnal variation. Clearblue Easy™ fertility monitors (Inverness Medical, Waltham, MA) aided in scheduling visits to appropriate menstrual cycle phases [25]. Almost all women (94%) provided at least 7 blood samples for PON1 activity analysis during each menstrual cycle; all women provided at least 5 blood samples per cycle. Total serum lipids (including: total cholesterol, HDL, and triglycerides) were measured in serum from each clinic visit using a Beckman LX20 automated chemistry analyzer with a CV of <5%. The Friedwald formula was used to indirectly determine LDL. F_2_-8α isoprostanes (isoprostane) and 9-hydroperoxy-10, 12-octadecadienic acid (9-HODE) were measured in EDTA (15% K_3_EDTA) anticoagulated plasma. Isoprostane levels in samples were determined by gas chromatography-mass spectrometry at the Molecular Epidemiology and Biomarker Research Laboratory of the University of Minnesota using an internal standard [26]. Total plasma 9-HODE was measured at the Oxidative Stress Research Laboratory of the University at Buffalo by high performance liquid chromatography with diode array detection at 234 nm following mild alkaline hydrolysis of lipid esters to yield total free fatty acids [27].

### Metals Exposure Analysis

Whole blood was collected at the screening visit in collection tubes (prescreened for trace metals) containing EDTA provided by the Centers for Disease Control and Prevention (CDC). Samples were analyzed for concentrations of cadmium, lead, and mercury by inductively coupled plasma mass spectrometry (ICP-MS) at the Division of Laboratory Sciences, National Center for Environmental Health, at the CDC. The limits of detection (LOD) for cadmium, lead, and mercury were 0.20 µg/dL (26.8% <LOD), 0.25 µg/dL (0% <LOD), and 0.30 µg/dL (10.8% <LOD), respectively. Values reported by the laboratory below the LOD were not substituted to minimize potential bias [28]. The interassay coefficients of variation for cadmium, lead, and mercury were 4.3%, 2.6% and 3.2% at levels of 2.04 µg/dL, 2.89 µg/dL, and 5.77 µg/L, respectively.

### PON1 activity and phenotype assignment

PON1 activity was measured in all blood samples collected during study clinic visits. PON1 activities and Q192R phenotype were determined on the Cobas Fara II chemistry analyzer as previously described [29]. PON1 arylesterase activity (PON1A) was determined as the rate of formation of phenol at 270 nm using 1 mmol phenylacetate substrate in 20 mM TRIS–HCl, pH 8.0, with 1.0 mM CaCl_2_ on the COBAS FARA II chemistry analyzer; the interassay coefficient of variation (CV) was <5%. PON1 paraoxonase activity (PON1P) was determined as the rate of formation of p-nitrophenol at 405 nm using 1 mmol/L paraoxon in 50 mmol/L glycine buffer, pH 10.5, with 1.0 mmol/L CaCl2, with or without 1 mol/L NaCl.

To discriminate between PON1 phenotypes we calculated activity ratios [30], [31]. The salt stimulation ratio (SALT/PA) was defined as the salt stimulated paraoxonase activity (SALT) over arylesterase activity using phenylacetate (PON1A) as substrate [24]. The inhibition ratio ((IA-IA_0_)/NIA) was defined as the phenylacetate inhibited arylesterase activity (IA) using p-nitrophenyl acetate as substrate minus the estimate of influence of non-specific arylesterase activity of other carboxylic ester hydrolases (IA_0_) divided by the non-inhibited arylesterase activity (NIA) using p-nitrophenylacetate alone as substrate [26]. Finally, a double ratio, dubbed the PON salt stimulation/similar substrate inhibition (PON 4SI) ratio, was defined as (SALT/PA)/((IA- IA_0_)/NIA).

PON1 phenotype was assigned based on the ratio of PON1 enzyme activities using these different analytical conditions and substrates and is defined as (salt stimulated PON1P)/PON1A)/(IA-IA_0_/NIA) where IA_0_ is the estimate of influence of non-specific arylesterase activity of other carboxylic ester hydrolases. This activity ratio phenotype has been proven to be 100% accurate in assigning PON1 Q192R phenotype in comparison to *Alw1* restriction fragment length polymorphism (i.e., genotype determination) [29]. Therefore, the term “phenotype” used throughout the present report represents the assignment given based on the procedure described above and is expected to concur with the participants' actual genotype with 100% accuracy. The non-salt stimulated and non-inhibited activities of PON1; PON1A and PON1P, are the activities reported here.

### Statistical Methods

Demographic characteristics were compared by tertile of blood cadmium, lead, and mercury concentration separately, and associations were assessed using Fisher's exact and ANOVA tests. PON1 activity values were log-transformed before analysis. Comparison of PON1 activity levels utilized repeated measures ANOVA tests with Bonferroni adjusted p-values for multiple comparisons. Linear mixed models were used to evaluate the association between blood metal concentrations and PON1 activities. These models accounted for non-independence between measurements and multiple cycles per woman. A first-order autoregressive moving-average structure was specified for the correlation matrix. Random intercepts accounted for variation in baseline antioxidant levels between women. Models were run separately by metal type, with the lowest metal tertile as the reference group. All models were evaluated 1) unstratified overall and 2) stratified by PON1 phenotype to account for different activity by phenotype. Percent change in PON1 activity levels was calculated by exponentiating the beta coefficient from the linear mixed models, subtracting 1 and multiplying by 100.

Confounder selection was determined by a review of the literature and by identifying associations with metal levels in our data. The covariates included in the adjusted models were age (continuous), BMI (continuous), smoking status (current/not current) and race (white, black, Asian, other). Cadmium models were additionally adjusted for leafy green vegetable servings (continuous) from the FFQ, and mercury and lead models were adjusted for fish (continuous) and shellfish (continuous) servings. Baseline FFQ and first cycle visit serum cholesterol measures were only available for 248 women.

Additionally, PON1 paraoxonase and mercury models were adjusted for same day isoprostane and 9-HODE to explore whether lipid peroxidation explained part of the relationship for reduced PON1 paraoxonase activity. As an additional sensitivity analysis, composite tertiles models were generated where a subject was categorized as high if her blood metals concentrations were in the highest tertile for at least two metals, and low if they were in the lowest tertile for at least two metals and otherwise was in the middle category. A sensitivity analyses restricting to nonsmokers, due to the low smoking prevalence was conducted. Statistical significance was defined as p<0.05, and all statistical analyses were conducted in SAS 9.3 (SAS Institute, Cary, NC).

## Results

### Subject characteristics and PON1 phenotype

Median (IQR) cadmium, lead, and mercury levels were 0.30 (0.19–0.43) µg/L, 0.87 (0.68–1.20) µg/dL, and 1.15 (0.58–2.00) µg/L, respectively. Participant characteristics are shown according to tertile of cadmium, lead, and mercury (Table 1). Older age was associated with higher cadmium and lead concentrations. Metal levels were associated with race, Asian women were over-represented in the highest tertile (T3) groups for all three metals. Cadmium and lead were significantly associated with race and whites were more likely to have lower levels of both metals, while those identifying as Asian, non-Hispanic Black, or other race were more likely to have higher levels. Although not significantly associated with race, Asian women were more likely to be in the upper tertile of mercury exposure. Further, increasing servings of fish and shellfish were associated with increasing lead and mercury levels. Cadmium was positively associated with smoking. Concentrations of cadmium, lead, and mercury were similar across the three PON1 phenotype groups (ANOVA Type III p-values = 0.59, 0.56, 0.13, respectively).

**Table 1:** Characteristics, diet, and serum cholesterol levels BioCycle Study participants, stratified by tertile of blood cadmium, lead and mercury.

			Cadmium Tertiles (µg/L)							Lead Tertiles (µg/dL)							Mercury Tertiles (µg/L)						
Characteristic	All		Lowest (mean = 0.15)		Middle (mean = 0.29)		Highest (mean = 0.61)		P	Lowest (mean = 0.58)		Middle (mean = 0.87)		Highest (mean = 1.63)		P	Lowest (mean = 0.39)		Middle (mean = 1.15)		Highest (mean = 2.87)		P
			0 -≤0.22		0.22 -≤0.36		>0.36			0 -≤0.72		0.72 -≤1.0		>1.0			0 -≤0.8		0.8 -≤1.6		>1.6		
N	250	100.0	80	100.0	82	100.0	88	100.0		78	100.0	90	100.0	82	100.0		82	100.0	79	100.0	89	100.0	
Mean Age (±SD)	27.4	8.2	25.9	7.9	26.8	7.7	29.4	8.7	0.01	25.5	6.8	28.0	9.0	28.6	8.3	0.05	26.2	8.4	28.7	8.1	27.3	8.2	0.16
Race (n(%))									<0.01							<0.01							0.09
White	149	59.6	60	75.0	49	59.8	40	45.5		57	73.1	54	60.0	38	46.3		53	64.6	48	60.8	48	53.9	
Asian	35	14.0	3	3.8	13	15.9	19	21.6		5	6.4	5	5.6	25	30.5		8	9.8	6	7.6	21	23.6	
Black	51	20.4	15	18.8	15	18.3	21	23.9		13	16.7	23	25.6	15	18.3		16	19.5	20	25.3	15	16.9	
Other	15	6.0	2	2.5	5	6.1	8	9.1		3	3.8	8	8.9	4	4.9		5	6.1	5	6.3	5	5.6	
Mean Body Mass Index (kg/m^2^) (±SD)	24.1	3.9	24.2	3.8	24	4	24.3	3.9	0.91	24.5	3.7	23.8	3.9	24.2	4.0	0.51	24.4	3.9	24.2	4.2	23.9	3.6	0.71
Smoking (n(%))									0.03							0.22							0.47
Nonsmoker	240	96.0	80	100.0	79	96.3	81	92.0		76	97.4	88	97.8	76	92.7		80	97.6	74	93.7	86	96.6	
Current smoker	10	4.0	0	0.0	3	3.7	7	8.0		2	2.6	2	2.2	6	7.3		2	2.4	5	6.3	3	3.4	
Kilocalories per day (±SD)^a^	1626.0	784.0	1664.9	796.8	1665.9	776.9	1554.3	782.9	0.57	1637.5	649.0	1682.3	866.4	1553.0	811.0	0.56	1571.3	763.6	1623.4	755.2	1678.1	831.1	0.68
Dietary iron, mg per day (±SD)^a^	12.6	7.2	12.9	7.0	13.4	8.7	11.7	5.8	0.27	12.8	6.3	13.3	7.0	11.7	8.2	0.37	12.2	8.3	12.8	7.1	12.9	6.4	0.78
Liver servings/6 months (±SD)^a^	7.5	33.9	5.5	31.1	5.6	25.8	11.1	41.9	0.48	6.4	26.8	12.9	48.8	2.7	13.2	0.13	3.3	20.9	10.6	42.7	8.7	34.7	0.36
Fish servings/6 months (±SD)^a^	34.0	67.2	37.9	56.4	35.9	96.1	28.8	37.5	0.65	21.5	30.4	35.1	48.5	44.8	100.9	0.09	12	18.2	28.1	33.7	59.1	101.3	<0.001
Shellfish/6 months (±SD)^a^	11.0	25.5	10.7	17.2	13.4	39.4	9.0	12.1	0.53	6.4	11.1	10.1	16.4	16.5	39.3	0.04	3.8	9.7	6.9	10.4	21.1	38.5	<0.001
Greens/6 months (±SD)^a^	292.4	263.5	251.4	243.5	302.2	271.4	320	271.8	0.23	276.2	274.3	299.9	264.7	299.6	254.0	0.81	263.4	256.9	290.4	256.9	320.4	274.9	0.37
Mean total cholesterol, mg/dL (±SD)^b^	163.7	28.9	161.0	26.4	162.9	27.5	166.8	32.1	0.42	160.9	24.9	162.6	25.7	167.6	35.0	0.31	164.1	26.5	161.5	29.9	165.3	30.3	0.69
Mean HDL cholesterol, mg/dL (±SD)^b^	50.1	11.6	50.1	11.6	49.6	12.7	50.5	10.7	0.89	47.5	11.0	50.9	10.3	51.6	13.2	0.06	50.8	10.6	48.3	10.5	51.0	13.4	0.27
Mean triglycerides, mg/dL (±SD)^b^	58.4	27.4	57.3	27.6	61.3	24	56.8	30.1	0.51	62.9	29.0	56.3	26.4	56.5	26.7	0.22	58.1	26.5	61.1	33.6	56.4	21.3	0.54

a Dietary information from baseline food frequency questionnaire was available for 248 women.

b Lipid profiles for 248 women performed on Day 2 of Cycle 1.

Categorical p-values: Fisher's exact. Continuous p-values: ANOVA.

**Figure 1:**
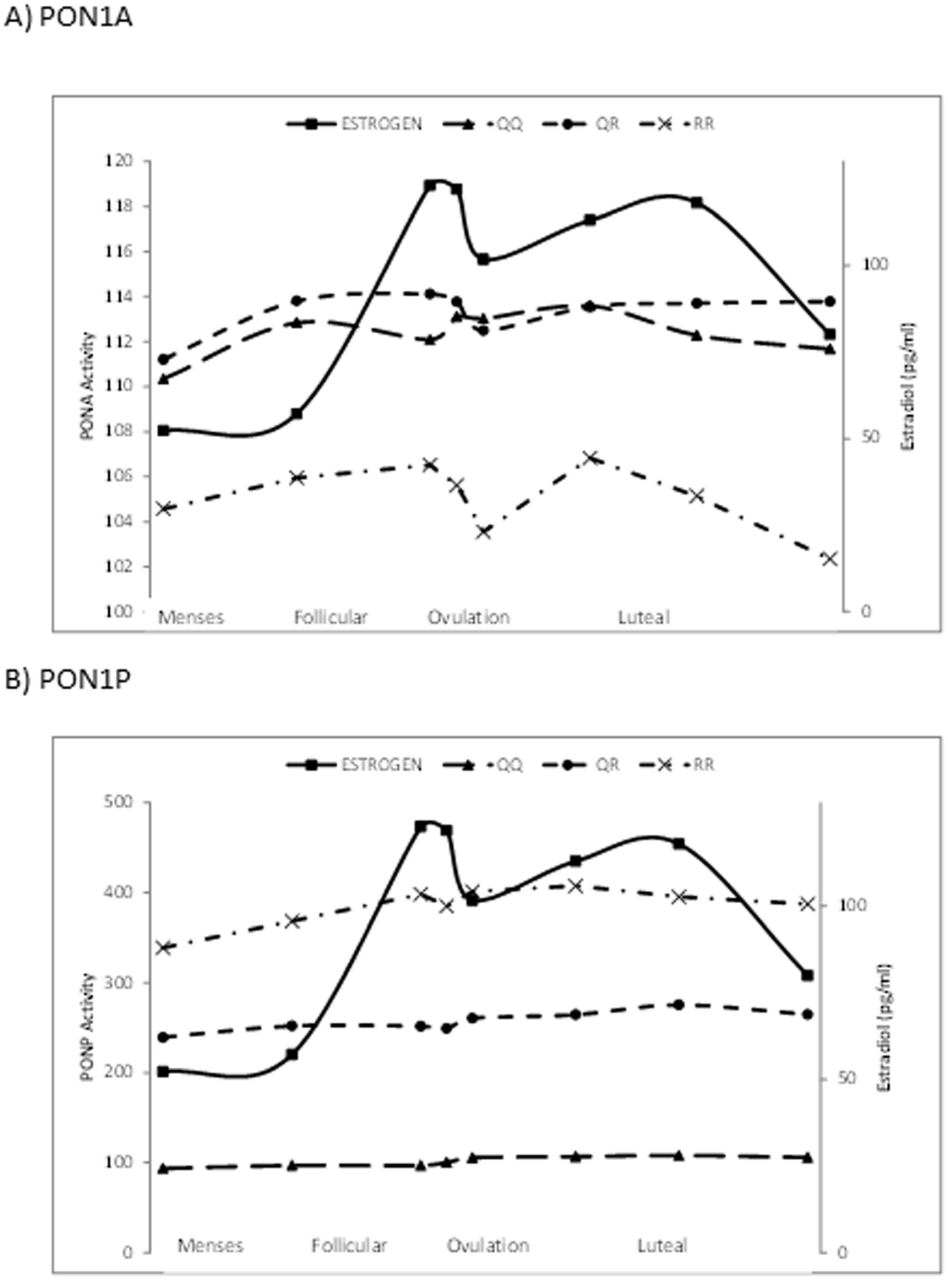
Geometric mean concentrations of estradiol, PON1A and PON1P across the menstrual cycle (N = 497 cycles) by PON1 phenotype.

In total, 3082 biospecimens were measured for PON levels. Most women had the PON1 QR (n = 117) phenotype, followed by QQ (n = 99), and RR (n = 34) (Table 2). Regarding differences across phenotypes, PON1A activity was greater in QQ (median activity 112.3 µmol/min/L) and QR (113.3 µmol/min/L) women compared to RR (105.1 µmol/min/L) women (p<0.0001 for both). PON1P activity was higher in RR (median activity 384.3 µmol/min/L) vs. QR (256.5 µmol/min/L) women, QR vs. QQ (101.4 µmol/min/L) women and in RR vs. QQ women (p<0.0001 for each). Geometric mean PON levels differed by phenotype. Non-Hispanic black women were more likely to have the RR phenotype (35%) and and less likely to have QQ, while non-Hispanic white women were less likely to have RR phenotype (5%) compared to the QQ (54%) and QR (41%) phenotypes. Within phenotype, PON1A activity varied across the menstrual cycle: for QQ phenotype, activity levels during the follicular phase were lower than in the luteal phase, and for QR phenotype, activity levels were lower during menses and around ovulation in comparison to the luteal phase (all Bonferroni adjusted p-values<0.10; Figure 1A) and for those with the QQ phenotype, activity decreased around ovulation. However, PON1P did not vary significantly across the menstrual cycle (Figure 1B).

**Table 2:** Geometric mean of PON1A and PON1P levels by metal tertile and genotype, in the BioCycle Study (N = 250), Buffalo, NY (2005–2007).

		QQ (n = 99)				QR (n = 117)				RR (n = 34)			
PON1A		Mean	SD	Min	Max	Mean	SD	Min	Max	Mean	SD	Min	Max
Cadmium	Highest (T3)	112.7	1.2	74.0	153.2	115.0	1.2	70.5	163.0	108.4	1.2	77.7	139.4
	Middle (T2)	112.9	1.2	83.7	152.0	103.0	1.3	62.4	168.3	86.3	1.5	42.3	142.5
	Lowest (T1)	111.3	1.2	60.1	159.7	122.1	1.2	77.9	151.3	118.0	1.3	63.4	148.3
Lead	T3	113.1	1.2	79.5	144.6	115.6	1.2	79.2	168.3	96.2	1.3	51.1	142.7
	T2	111.5	1.2	60.1	153.2	111.3	1.2	62.4	159.2	101.1	1.5	42.3	143.1
	T1	112.6	1.2	74.0	159.7	112.3	1.3	68.3	163.0	129.7	1.2	99.2	148.3
Mercury	T3	118.3	1.2	79.5	153.2	112.3	1.3	68.3	168.3	97.1	1.5	42.3	145.8
	T2	107.4	1.2	60.1	151.2	110.7	1.2	62.4	159.2	101.1	1.4	63.4	146.3
	T1	111.1	1.2	80.1	159.7	117.3	1.2	82.9	148.7	117.7	1.2	96.4	148.3
PON1P													
Cadmium	T3	109.3	1.4	50.1	239.6	270.7	1.3	153.5	424.9	393.0	1.2	249.9	497.9
	T2	104.1	1.4	57.0	216.0	233.8	1.3	130.2	438.9	316.8	1.6	129.3	611.7
	T1	90.7	1.6	39.6	213.1	263.0	1.3	129.7	438.7	431.2	1.3	269.5	658.1
Lead	T3	95.3	1.5	45.2	183.2	250.7	1.3	141.3	438.7	355.7	1.3	196.4	518.8
	T2	104.9	1.5	39.6	239.6	259.6	1.3	129.7	438.9	379.0	1.6	129.3	611.7
	T1	102.1	1.4	52.2	189.2	258.8	1.3	153.5	426.8	449.8	1.2	314.7	658.1
Mercury	T3	101.0	1.6	39.6	239.6	263.1	1.3	141.3	438.7	354.8	1.5	129.3	518.8
	T2	89.8	1.4	45.2	183.2	249.4	1.4	129.7	426.8	364.7	1.4	196.4	658.1
	T1	111.6	1.4	50.1	216.0	255.7	1.3	166.2	438.9	433.0	1.2	314.7	611.7

### Relationship between PON1 enzyme activity and metals exposure

PON1A activity (Table 3) was 10.9% (95% confidence interval [CI] −16.5%, −4.8%) lower and 7.1% (−13.0, −0.8) lower for women with the middle tertile (T2) of cadmium and mercury. Lead was not associated with a significant change in PON1A levels and there was no evidence of a dose response for the upper tertile (T3) of cadmium and mercury. Without stratifying by phenotype, PON1P was not associated with metal levels. After stratifying by phenotype, PON1A activity (Table 3) was 27.9% and 14.4% lower among women with middle tertile (T2) blood cadmium levels compared to low levels for RR (95% confidence interval [CI] −39.5%, −14.0%) and QR (95% CI −20.1%, −8.4%) phenotypes. PON1A activity was 6.5% lower among women with the QR phenotype among women with T3 cadmium levels (95% CI −12.7, 0.0). Similarly, PON1P activity was 36.9% and 9.5% lower among those with T2 cadmium levels for RR (95% CI −48.0%, −23.5%) and QR (95% CI −18.1, 0.0) compared to low levels. Conversely, among those with the QQ phenotype, cadmium was associated with a 24.5% increase in PON1P (95% CI 7.0, 44.9). Consistent results with respect to cadmium were noted after restricting to nonsmokers.

**Table 3:** Percent change in PON1A and PON1P activity levels overall and by tertile of blood metal level, in the BioCycle Study (N = 250), Buffalo, NY (2005–2007).

Metal	Model^a^	Tertile	Unadjusted overall percent change (95% confidence interval)	Adjusted overall percent change (95% confidence interval)	QQ n = 99	Percent change (95% confidence interval)	QR n = 115^b^	Percent change (95% confidence interval)	RR n = 34	Percent change (95% confidence interval)
Cadmium	PON1A	T3^c^	−2.2 (−8.4, 4.4)	−4.0 (−10.4, 2.9)	32	1.1 (−5.9, 8.7)	45	−6.5 (−12.7, 0.0)	11	−3.7 (−17.2, 11.9)
		T2	−10.3 (−16.0, −4.3)	−10.9 (−16.5, −4.8)	36	2.3 (−3.7, 8.7)	36	−14.4 (−20.1, −8.4)	9	−27.9 (−39.5, −14.0)
		T1	Reference	Reference	31	Reference	34	Reference	14	Reference
	PON1P	T3	7.2 (−11.4, 29.7)	−4.1 (−20.6, 15.9)	32	24.5 (7.0, 44.9)	45	2.4 (−7.3, 13.2)	11	−7.0 (−21.2, 9.8)
		T2	−11.8 (−27.1, 6.7)	−16.4 (−30.2, 0.0)	36	17.9 (3.6, 34.2)	36	−9.5 (−18.1, 0.0)	9	−36.9 (−48.0, −23.5)
		T1	Reference	Reference	31	Reference	34	Reference	13	Reference
Lead	PON1A	T3	−3.2 (−9.6, 3.5)	−4.2 (−10.8, 2.8)	26	−6.4 (−12.8, 0.5)	40	3.9 (−2.9, 11.1)	15	−18.9 (−32.5, −2.5)
		T2	−3.8 (−9.8, 2.7)	−4.8 (−10.8, 1.6)	37	−1.7 (−7.5, 4.4)	42	−1.0 (−7.1, 5.6)	10	−19.6 (−32.4, −4.4)
		T1	Reference	Reference	36	Reference	33	Reference	9	Reference
	PON1P	T3	6.9 (−12.3, 30.3)	−3.2 (−20.2, 17.5)	26	−10.3 (−32.9, 19.8)	40	1.2 (−16.6, 22.8)	15	−1.6 (−31.8, 41.8)
		T2	2.2 (−15.5, 23.5)	−4.5 (−20.1, 14.2)	37	6.4 (−9.8, 25.6)	42	0.8 (−10.6, 13.7)	10	−11.7 (−31.5, 13.8)
		T1	Reference	Reference	36	Reference	33	Reference	13	Reference
Mercury	PON1A	T3	−1.0 (−7.1, 5.6)	0.3 (−6.1, 7.3)	33	6.2 (0.2, 12.6)	44	−2.8 (−9.6, 4.5)	12	6.3 (−10.0, 25.6)
		T2	−6.0 (−12.0, 0.4)	−7.1 (−13.0, −0.8)	29	−5.8 (−11.5, 0.3)	40	−4.2 (−10.6, 2.6)	9	−11.7 (−24.7, 3.6)
		T1	Reference	Reference	37	Reference	31	Reference	13	Reference
	PON1P	T3	−1.3 (−18.2, 19.0)	−1.8 (−18.2, 18.0)	33	−12.2 (−28.0, 7.1)	44	5.6 (−11.2, 25.6)	12	−12.9 (−34.8, 16.3)
		T2	−8.7 (−24.6, 10.6)	−11.3 (−25.9, 6.1)	29	−25.7 (−37.2, 11.9)	40	−2.3 (−13.9, 10.8)	9	−22.8 (−37.8, −4.1)
		T1	Reference	Reference	37	Reference	31	Reference	13	Reference

a Adjusted for age (continuous), race (white, black, Asian, other), BMI (continuous), smoking (current, no), leafy green servings (continuous), fish servings (continuous), shellfish servings (continuous), concurrent metal levels (tertiles).

b 115 women with diet information were available for the adjusted models.

c T3: Highest, T2: Middle, T1: Lowest.

With respect to lead levels, among women with RR phenotype, PON1A activity was diminished for those in T3 (−18.9%, 95% CI −32.5%, −2.5%) and T2 (−19.6%, 95% CI −32.4%, −4.4%). Lead was not statistically significantly associated with PON1P activity.

Regarding mercury exposure, PON1P activity was lower in T2 (−22.8%, 95% CI −37.8%, −4.1%) compared to those with T1 among the RR phenotype. This association was unaffected by further adjustment for isoprostane and 9-HODE, respectively (data not shown). Among those with the QQ phenotype, mercury was associated with a statistically significant 6.2% increase in PON1A (95% CI 0.2, 12.6).

Interestingly, the majority of associations observed between PON1 enzyme activity and metal exposure were attributable to differences at the middle tertile level of metal concentration relative to the lowest level, rather than the highest tertile compared to the lowest. After generating a composite metal category, among those with the RR phenotype, statistically significant decreases in PON1A and PON1P activities persisted for the middle tertile of exposure, although not when comparing the highest tertile to the lowest (Table 4).

**Table 4:** Percent change in PON1 arylesterase (PON1A) and PON1 paraoxonase (PON1P) activity levels by tertile of composite^a^ blood metal level, in the BioCycle Study (N = 250), Buffalo, NY (2005–2007).

	Model			QQ (n = 99)		QR (n = 117^b^)		RR(n = 34)
			n	% change	n	% change	n	% change
PON1A	A	T3^c^	23	0.4 (−6.4, 7.7)	38	−6.8 (−13.2, 0.1)	12	−19.0 (−30.7, −5.3)
		T2	46	−0.5 (−6.2, 5.6)	53	−16.3 (−21.7, −10.5)	11	−22.0 (−33.4, −8.6)
		T1	30	Ref	26	Ref	11	Ref
	B	T3	23	−4.7 (−11.9, 3.0)	38	−6.8 (−13.5, 0.5)	12	−7.9 (−22.4, 12.2)
		T2	46	−0.4 (−6.3, 5.9)	52	−15.3 (−20.6, −9.6)	11	−21.7 (−33.6, −7.6)
		T1	30	Ref	25	Ref	11	Ref
PON1P	A	T3	23	3.9 (−10.3, 20.4)	38	−3.0 (−12.4, 7.3)	12	−20.2 (−33.1, −4.8)
		T2	46	3.6 (−8.6, 17.3)	53	−10.5 (−18.7, −1.6)	11	−28.3 (−40.0, −14.2)
		T1	30	Ref	26	Ref	11	Ref
	B	T3	23	−5.0 (−20.0, 12.9)	38	−3.7 (−14.1, 8.0)	12	−14.2 (−32.7, 9.4)
		T2	46	−1.2 (−13.6, 13.0)	52	−10.3 (−18.7, −1.0)	11	−28.8 (−41.8, −12.9)
		T1	30	Ref	25	Ref	11	Ref

a Composite tertiles definitions: high (in highest tertile of at least two metals), low (in lowest tertile of at least two metals), medium (else).

b There were only 115 women with diet information available for the adjusted models.

c T3: Highest, T2: Middle, T1: Lowest.

A: unadjusted.

B: adjusted for age (continuous), race (white, black, Asian, other), BMI (continuous), smoking (current, no), leafy green servings (continuous), fish servings (continuous), shellfish servings (continuous), concurrent metal levels (tertiles).

## Discussion

Considering all phenotypes together, PON1P was not associated with metal levels while the middle tertiles of cadmium and mercury were associated with decreased PON1A, but there was no dose-response relationship evident. After stratifying by phenotype, women with the RR phenotype exhibited some attenuated PON1A and PON1P enzyme activity in association with increased blood cadmium and lead concentration. However, the dose-response relationship was not linear and the middle category of cadmium and lead was consistently associated with a greater reduction in enzymatic activity compared with the greatest exposure category. Such evidence suggests that nonessential metals exposure could be associated with PON1 enzyme activity in healthy young women; however, given the number of statistical tests conducted and small sample size, such findings may be attributable to chance and await replication. Overall, these data provide limited support that PON1 phenotype could play a role in determining PON1 functional susceptibility to cadmium and lead exposure, even at relatively low background levels. The combination of PON1 phenotype and environmental cadmium and lead exposure may play a role in an individual's risk for cardiovascular disease. Further, PON1A activity varied across the menstrual cycle by phenotype, which has not been previously reported. We found significant racial differences in PON phenotype. Specifically, non-Hispanic blacks had a higher proportion of RR phenotype compared with whites, which was previously noted among a population sample from the southern US [32]. Among women with the QQ phenotype, cadmium and mercury were associated with increases in PON1P levels.

PON1A and PON1P activities significantly modulate systemic oxidative stress and cardiovascular disease risk [1], [33]. In a large (approx. 1,400) prospective study of patients undergoing diagnostic coronary angiography, a lower risk of all-cause mortality and major acute cardiac events was observed for patients with RR and QR phenotypes, but decreased PON1 enzyme activity was a stronger determinant of more rapid onset of an acute major cardiac event than PON1 phenotype alone [1]. Our finding that women with the RR phenotype had diminished PON1 enzyme activity in association with cadmium and lead exposure may imply that the relationship between Q192R phenotype and enzyme activity vary by sex and by chronic disease status. The study population in Bhattacharyya et al. was undergoing cardiac screening and many had prevalent disease, which could change the PON1 activity in comparison to young, healthy women. These findings should be interpreted with caution as only 34 participants in the BioCycle Study had the RR phenotype and these findings await corroboration. Others indicated that PON1 enzyme activity, versus phenotype alone, may be a more meaningful marker of atherosclerosis and coronary heart disease [33], [34]. Collectively, such evidence suggests that non-genetic modulators of PON1 enzyme activity may also modulate cardiovascular disease and mortality risks through their effects on PON1. Indeed, a recent trial of lycopene supplementation, a dietary antioxidant, in overweight subjects indicated that one mechanism by which lycopene helps reduce cardiovascular morbidity may be through increasing PON1 activity [35].

In the present study, we evaluated the role of environmental metals in association with PON1 functionality. Women with the RR phenotype had the strongest decrease in PON1 enzyme function (approximately 30% reduction), associated with blood cadmium concentrations. PON1A activity was attenuated in the QR phenotype, but by nearly half of the effect observed in RR women. Meanwhile, among those with the QQ phenotype, PON1A activities were not associated with cadmium, but PON1P activities were increased. Thus, cadmium exposure, even at the low levels observed in this cohort of healthy women, may play a role in diminishing the cardio-protective function of PON1, particularly among those with either RR or QR phenotype. Of note, PON activity remains higher among RR or QR phenotype compared with QQ, despite the observed associations with metals. The deleterious impact of cadmium on PON1 enzyme activity suggests that suppression of PON1 may be a mechanism by which smoking impairs cardiovascular health as cigarette smoking is a major contributor to cadmium exposure [36]. Studies in rodents indicate that cadmium increases PON1 activity following acute exposure, but extended exposure results in diminished antioxidant capacity and greater lipid peroxidation [37]. These data indicate a (time and/or dose) limit after which the capacity for PON1 in neutralizing the impact of environmental oxidative toxicity may diminish, and PON1 activity declines relative to increasing total cadmium exposure, as observed here.

Elevated blood lead levels were associated with impaired PON1 function in women with RR phenotype. Notably, this occurred despite the median lead concentration in this cohort of women (0.87 µg/mL) being >10 times lower than the U.S. Centers for Disease Control (CDC, 10 µg/mL) level of concern in children, which was recently recommended to be revised given a range of health effects observed below this level [38]. In a study of 597 workers with occupational lead exposure (having blood lead levels more than 30 times the level in the present study) [39], the impairment of PON1 enzyme function effect was also greatest in those with the RR PON1 phenotype. We observed a significant association between lead and PON1 arylesterase activity among those with the RR phenotype which is similar to previous occupational studies on PON1 arylesterase function and lead exposure [39].

Few previous reports have examined the effect of mercury exposure on PON1 enzyme activity [40], but one report indicated a negative effect of methylmercury on PON1 function in an Inuit population without detecting variation in this effect by PON1 phenotype. However, this previous study evaluated only one enzymatic function of PON1 (organophosphatase activity), had incomplete phenotype data and did not report details regarding covariate inclusion in statistical models [41].

Strengths of the present investigation include the direct measurement of blood metal concentrations and PON1 enzyme activity in a homogenous, well-defined cohort of healthy premenopausal women to evaluate background metals exposure. Multiple measurement of PON1 enzyme activity across two menstrual cycles allowed for evaluation of variability across the cycle and added to precision. Variability of PON1A across the menstrual cycle has not previously been reported and this may be related to endogenous reproductive hormones, although this finding awaits corroboration. That similar variability was observed for PON1P is not known but may be related to the enzyme substrate. A limitation of this study is that the metals were measured at only one time point and in blood. However, blood levels are considered a stable reflection of exposure for the time period of the study (2 months). Further, the ability to measure and account for dietary sources of these nonessential metals was a strength as this represents an important exposure source. While the metal exposure level in this cohort differs somewhat compared with other populations, unless one expects the relationships to differ significantly by exposure level, our results remain generalizable. Further, the median blood concentrations of cadmium, lead and mercury detected in this study were comparable to those reported in other populations having only non-occupational exposure to such metals, and as such are directly relevant to our understanding of effects at environmentally relevant levels of exposure [9]. BioCycle Study participants had higher mercury levels than reproductive-aged US women and comparable lead and cadmium levels [42]. Another perceived limitation is the use of enzyme activity measurement to assigned PON1 phenotype, as opposed to direct genotyping; however, the method used here to assign PON1 phenotype by combining information from five different PON1 activity assays has been shown to have 100% efficiency in accurately separating the three phenotypes compared to genotyping by PCR [29]. The exclusion of postmenopausal women, premenarcheal girls, and men from the present study is another limitation as it is not possible to know if our findings extend to these important population groups. An additional limitation is the measurement of metal at baseline only. This precluded an analysis of the association between metals and levels of PON1A and PON1P during different menstrual cycle phases. Measurement of metals at different phases of the menstrual cycle would be necessary to facilitate such analysis. Finally, it is unknown why the majority of significant effects reported here were observed at middle tertile levels of exposure. To further investigate this phenomenon, we developed a summary measure of metals exposure, which confirmed our previous finding with respect to stronger decreases among those in the middle, rather than the highest category of exposure. These findings remain to be corroborated.

In conclusion, considerable decreases in PON1 function occurred in otherwise healthy women with background levels of exposure to cadmium and lead. However, the majority of such associations were observed when comparing T2 to T1, rather than T3 to T1. Therefore, such findings should be interpreted with caution due to the lack of consistent dose-response relationship. Given the established role of PON1 as a cardio-protective enzyme carried by circulating HDL, although consistent dose-response relationships were not observed in this study, we posit that nonessential metals exposure may play a role in ongoing cardiovascular disease risk, via antioxidant (such as PON1) function. However, no evidence of overall impaired lipid peroxidation was found in this population [20]. Importantly, the susceptibility of PON1 to cadmium and lead was dependent on individual PON1 phenotype, with the rarer form of RR demonstrating the most impairment and the more prevalent heterozygote form of QR exhibiting somewhat lesser susceptibility. PON1 phenotype may represent the contribution that genetics makes towards determining individual cardiovascular disease risk. Future investigations are needed to determine the consistency of our findings in other populations. In addition, research on the effects of metals on chronic disease risk factors may consider PON1 phenotype to account for individual susceptibility to such exposure.
